# Dissolution Behavior of Hydrothermally Treated Hydroxyapatite–Titanium Nitride Films Coated on PEEK: In Vitro Study

**DOI:** 10.3390/jfb13030099

**Published:** 2022-07-19

**Authors:** Siriwat Boonpok, Kwanchanok Koonrungsrisomboon, Kullapop Suttiat, Piriya Yavirach, Dhreerawan Boonyawan

**Affiliations:** 1 Department of Prosthodontics, Faculty of Dentistry, Chiang Mai University, Chiang Mai 50200, Thailand; siriwat_b@cmu.ac.th (S.B.); kwanchanok_koon@cmu.ac.th (K.K.); piriyavdx-18@hotmail.com (P.Y.); 2 Plasma and Beam Physics Research Facility, Department of Physics & Materials Science, Faculty of Science, Chiang Mai University, Chiang Mai 50200, Thailand; dheerawan.b@cmu.ac.th

**Keywords:** polyetheretherketone (PEEK), pulse DC magnetron sputtering, hydroxyapatite–titanium nitride coating, dissolution

## Abstract

Polyetheretherketone (PEEK) has become an alternative material for orthopaedics and dental implants. However, bio-inertness is an important limitation in this material. In the present study, a hydroxyapatite (HA)–titanium nitride (TiN) coating was fabricated via pulsed DC magnetron sputtering and treated with hydrothermal treatment to improve the bioactive property of PEEK. The dissolution behavior of the coating was studied in simulated body fluid solution (SBF) at 1, 3, 5, 7, 14, 21, 28, and 56 days. The coating surface was analyzed before and after the immersion process by X-ray photoelectron spectroscopy (XPS), atomic force microscope (AFM), and scanning electron microscope (SEM). The calcium and phosphorus concentration alteration in SBF was quantified by an inductively coupled plasma-optical emission spectrometer (ICP-OES). Coating dissolution and the precipitation of calcium phosphate complex from SBF were observed as occurring suddenly and continuously throughout the immersion times. These processes resulted in an alteration in both physical and chemical coating properties. After 56 days, the coating remained on PEEK surfaces and the Ca/P ratio was 1.16. These results indicate that HA-TiN coating via pulsed DC magnetron sputtering followed by hydrothermal treatment improved the bioactivity of materials and provided a potential benefit to orthopedics and dental applications.

## 1. Introduction

Polyetheretherketone (PEEK) is a non-absorbable polymer with good mechanical properties and excellent biocompatibility [1]. PEEK has been used in various industries and has become an alternative material for medical and dental appliances [1,2,3,4,5] due to its many suitable properties, particularly the elastic modulus close to the human bone (Table 1). This characteristic reduces stress around the implant and leads to a lesser stress-shielding effect than metallic materials [2,3,4]. A previous study reported that PEEK cages were used for orthopedic implants [1] and can also be used as dental implants [5]. However, despite PEEK’s advantages, the lack of bioactive properties is one of the significant drawbacks of this material and the low surface energy, which affects cell adhesion and proliferation at the surface, resulting in decreased bone adhesion [3,6,7,8].

Several techniques have been introduced to improve the bioactivity of the PEEK surface, including the coating of bioactive materials [6,7,8]. From previous studies, titanium nitride (TiN) and hydroxyapatite (HA) were recommended as biomaterials for improving bioactivity in medical and dental materials [6,9,10,11]. The high hardness and the excellent biocompatibility of TiN can improve the corrosive and wear resistance of the materials [9,11]. Furthermore, the osteoconductive property, accepted as a crucial factor, plays a significant role in the bone healing process as the osteointegration of implanted materials was found in the HA particles [10,12,13,14]. Therefore, the combination of these two materials is considered a technique for improving both the physical and biological properties of the PEEK [15].

Pulsed DC magnetron sputtering is a coating technique that deposits the particles from the target by bombardment reaction toward the substrates [17,18]. The sputtering process provides thin, pure, and highly dense uniform deposited films [19,20]. Moreover, the substrates’ original properties and architecture, especially the heat-sensitive materials, can be maintained following this surface coating technique [20,21].

Previous studies showed that HA coating from the sputtering process had low crystallinity, leading to less dissolution resistance and low adhesion force to the substrate material [22,23,24]. Hydrothermal treatment is a post-treatment process using heat and water vapor to recrystallize. [24] Hydrothermal treatment has been introduced to improve the crystallinity of the HA coating [24,25,26]. Buranapanich et al., 2020 found that the hydrothermal treatment of HA coating at 100 °C for 12 h increased HA crystallinity and provided the Ca/P ratio close to the stoichiometric ratio (1.67) [27]. Another important concern is the dissolubility of the HA-TiN coating, since it influences the stability of the implants and the osseointegration process [28]. Furthermore, it may alter the characteristics of the coating and result in bone remodeling around the implant materials.

The present study evaluated the in vitro dissolution process of the hydrothermally treated HA-TiN coating on PEEK. The Ca/P ratio and characteristics of HA-TiN coating following the in vitro degradation test were observed in SBF at 37 °C at various immersion times.

## 2. Materials and Methods

### 2.1. Specimen Preparation

Twenty-seven disc-shaped PEEKs were milled from Pure PEEK (Ketron 1000 PEEK, Quadrant Engineering Plastic Products, Reading, PA, USA). The PEEK discs were 6 mm in diameter and 2 mm in thickness. PEEK discs were polished with waterproof abrasive paper and water by the specimen preparing machine (Mopao 160E, LaiZhou Weiyi Experimental Machinery Manufacture, Shandong, China). Then, PEEK discs were cleaned in ethanol followed by acetone with an ultrasonic machine. (Easyclean, Renfert, Hilzingen, Germany) A profilometer verified the roughness of the discs. (Surftest 310, Mitutoyo, Kawasaki, Japan).

### 2.2. Coating Procedures and Hydrothermal Treatment

**Figure 1:**
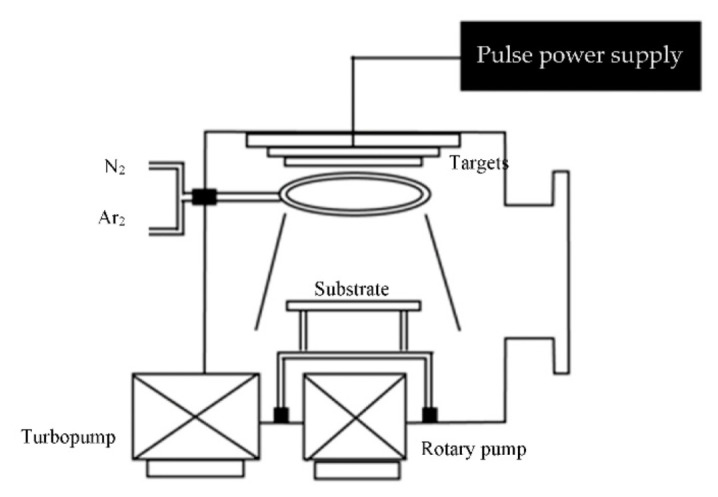
A diagram of pulsed DC magnetron sputtering chamber.

A pulsed DC magnetron sputtering machine with a 37 × 37 × 37 cm^3^ stainless rectangular steel chamber (Figure 1) was used for fabrication the HA-TiN on the PEEK. The 99.99% titanium (commercial grade) and HA disc were prepared. The commercial HA powder was mixed 2.8% w polyvinyl alcohol as the binder then pressed for 10 min and sintered at 1200 °C for 3 h. The titanium and HA disc were used as targets. The distance between the specimens and target was 8 cm. A turbomolecular and rotary pump was used to control the base pressure to less than 7.5 × 10^−6^ torr. A mixture of argon was used as the sputtering gas and nitrogen was used as the reactive gas with a flow rate of 3.5 and 1.4 sccm, respectively. The DC Generator (TruPlasma DC 4001, TRUMPF, Ditzingen, Germany) was the pulse power supply. The sputtering was performed at a pressure between 3.0 and 3.5 × 10^−2^ torr. The pulse voltage, frequency, and time were set as 400 V, 50 kHz, and 2 microseconds, respectively. The sputtering time was set for 4 h.

After the sputtering process, the specimens were treated with a hydrothermal treatment in a Teflon container filled with 10 mL of deionized water. The hydrothermal process was performed in an autoclave cabinet (Memmert BE 400, Memmert, Schwabach, Germany) at 100 °C for 12 h. The phase composition and crystallinity of coatings were identified by X-ray diffraction (XRD) [29] (Smartlab, Rigaku, Shibuya, Japan).

### 2.3. Specimen Immersion in Simulated Body Fluid (SBF)

SBF (SBF, Phygene Biotechnology, Fuzhou, China) has an ionic concentration similar to human body plasma, as shown in Table 2. In this study, SBF was used for the immersion test. The HA-TiN coated PEEK discs were divided into 9 groups (n = 3) according to the immersion time (1, 3, 5, 7, 14, 21, 28, and 56 days), wherein all groups were incubated at 37 °C. The specimens were rinsed with deionized water after the completed immersion test, dried, and kept for characteristic evaluation. The Ca and P amounts in SBF at each interval were quantified by an inductively coupled plasma-optical emission spectrometer (ICP-OES, Optima 7300 DV, PerkinElmer, Waltham, MA, USA).

### 2.4. Coating Characteristic Evaluation

The chemical alteration of the coating after the immersion in SBF was evaluated by X-ray photoelectron spectroscopy (XPS, AXIS Ultra XPS spectrometer, KRATOS analytical, Stretford, Manchester, UK). The parameters for XPS were set as in a previous study [27]; the monochromatic Al K X-ray was at 150 W anode power, survey spectra from 0 to 1200 eV with a pass energy of 80 eV for a survey, and 20 eV for core-level spectra. Two specimens of each group were used for the XPS test. Each specimen was tested for two points. In addition, the atomic percentage of each element was measured. The average value of calcium (Ca) and phosphorus (P) was used for the Ca/P ratio calculation.

The surface morphology was analyzed via scanning electron microscope (SEM) (JSM-IT300LV, JEOL USA Inc., Peabody, MA, USA). The coating surface roughness was evaluated by atomic force microscope (AFM) (Park XE7, Park system, Mannheim, Germany) in non-contact mode. The surface roughness measurement was repeated four times on each specimen. The average roughness value was calculated.

All experiments and tests were performed at Chiang Mai University, Thailand.

## 3. Results

### 3.1. Coating Characteristics

The coating characteristics were analyzed for chemical properties by using an XRD and XPS. In addition, the surface morphology was analyzed by AFM and SEM.

#### 3.1.1. Crystallinity of Coatings

**Figure 2:**
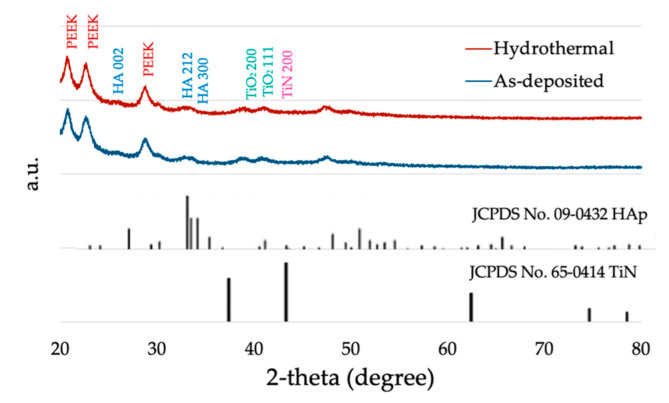
The XRD pattern of hydrothermally treated and as-deposited HA-TiN coating.

XRD patterns of the as-deposited and the hydrothermal-treated HA-TiN are presented in Figure 2 with the standard XRD pattern from the International Centre for Diffraction Data (ICDD) of HA (JCPDS No. 09-0432) and TiN (JCODS No. 65-0414). The XRD pattern of coatings shows broad base peaks of HA002, HA212, and HA300, which indicated that this coating was dominant in the amorphous phase of HA coating.

#### 3.1.2. Chemical Composition of Coatings

**Figure 3:**
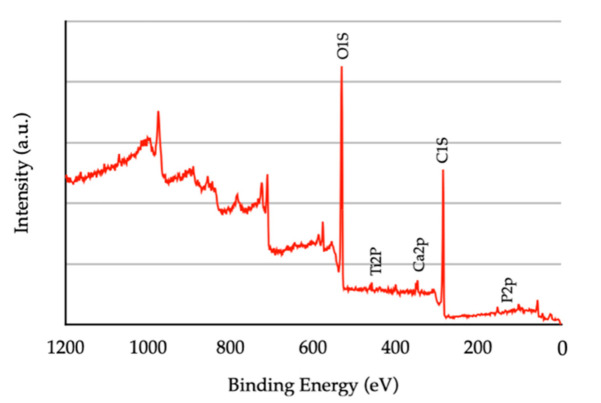
The XPS survey spectra of hydrothermal-treated HA-TiN coating layer. The observed peaks represent the oxygen and calcium atoms. A minor amount of titanium and phosphorus was also exhibited.

The XPS survey spectra of hydrothermally treated HA-TiN coatings are shown in Figure 3. The National Institute of Standards and Technology (NIST) standard reference database found the atoms of oxygen and carbon as significant components. In contrast, some amounts of calcium, phosphorus, and titanium were minor components.

**Figure 4:**
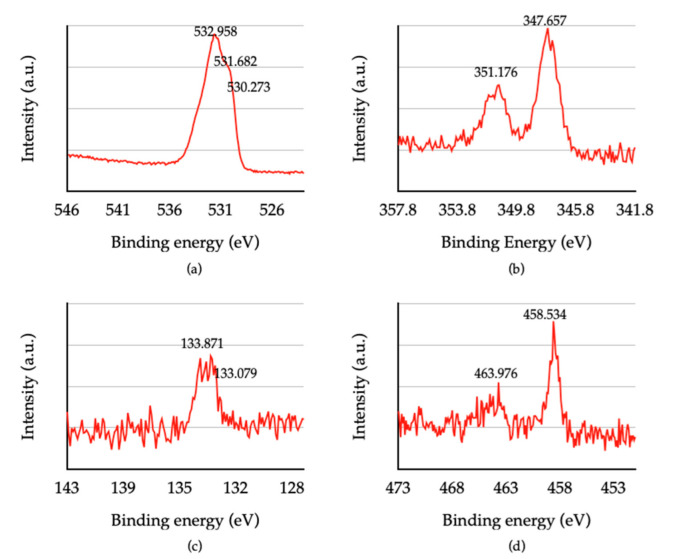
The narrow scanning XPS spectrum, (**a**) the high-resolution XPS spectra of O1s, (**b**) the high-resolution XPS spectra of Ca2p, (**c**) the high-resolution XPS spectra of P2p, (**d**) the high-resolution XPS spectra of Ti2P.

The narrow scanning XPS spectrum of O1s, Ca2p, and P2p peaks is shown in Figure 4. For the O1s peak, the three small peaks represented the oxide bond of titanium, phosphate, and other hydrocarbons, at a binding energy of 530.273 eV, 531.682 eV, and 532.985 V, respectively (Figure 4a). The expression of the phosphate bond in O1s peak is related to HA formation on the coating layer. The binding energies 347.657 eV and 351.176 eV, related to the HA, were also noticed on the Ca2p peaks (Figure 4b). The P2p peaks were divided into p1/2 and p3/2, with binding energy of 133.871 eV, which was inferred to be a phosphate bond in HA (Figure 4c). Although HA formation occurred, the formation of TiN could not be observed. Two Ti2p peaks were expressed in the formation of titanium dioxide (TiO_2_) instead of TiN (Figure 4d). The average atomic percentage ratio of Ca and P found in the hydrothermally treated HA-TiN coating was 1.284.

**Figure 5:**
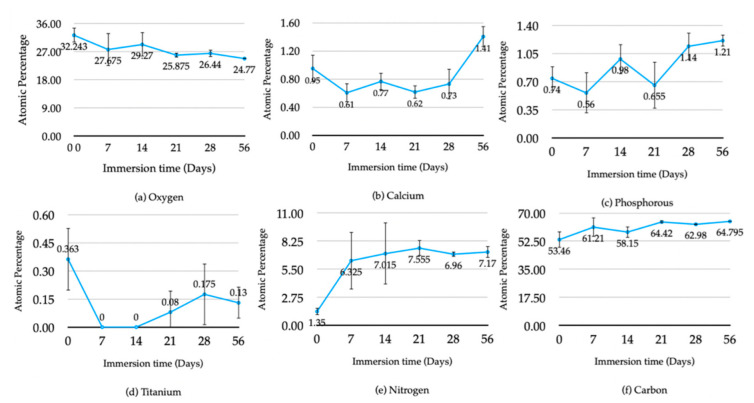
The Average atomic concentration of components of hydrothermally treated HA-Tin coating at various immersion times: (**a**) oxygen, (**b**) calcium (**c**), phosphorus, (**d**) titanium, (**e**) nitrogen, (**f**) carbon (error bar S = 1 SD).

In Figure 5, from the first to the third week of immersion, the average atomic percentage of oxygen, calcium, phosphorus, and titanium components of HA-TiN coatings showed fluctuating tendencies, resulting from the dynamic process of dissolution and precipitation. After the third week of immersion, the atomic percentage of oxygen continued to a decrease until the eighth week of immersion, while others increased, especially Ca and P.

**Figure 6:**
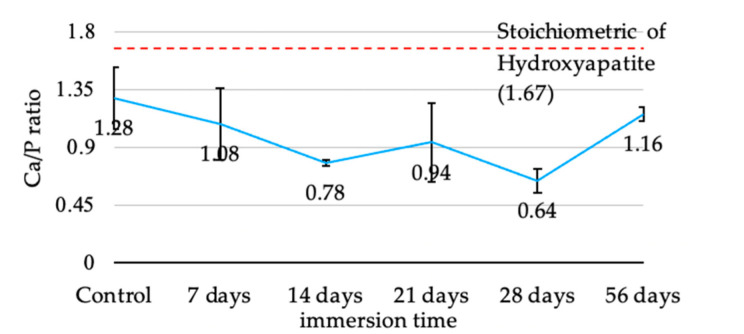
The average Ca/P ratio of hydrothermally treated HA-TiN coating at various immersion times (error bar = 1 SD).

The Ca/P ratio of the coating during immersion is shown in Figure 6. In the first stage in the first two weeks of immersion, the Ca/P ratio continuously decreased from 1.284 to 1.08. and 0.78 in the first and second week, respectively. Then, the Ca/P ratio stabilizes from the second to eighth week. After immersion for 56 days, the Ca/P ratio of coatings was 1.16.

#### 3.1.3. Surface Morphology of Coatings

**Figure 7:**
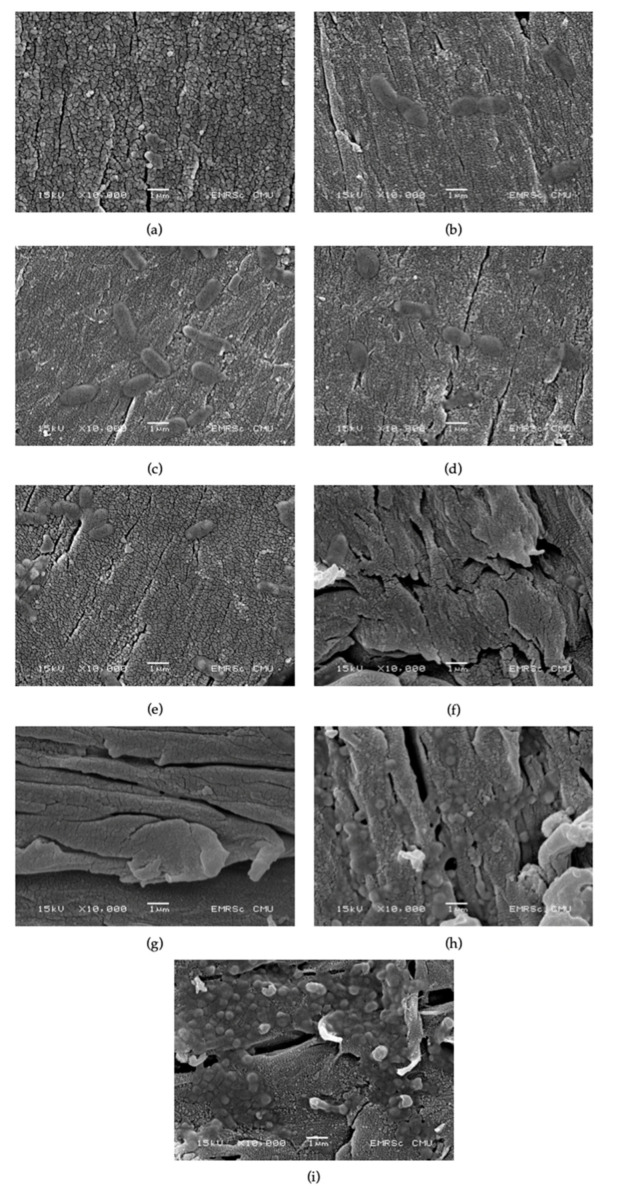
SEM images of hydrothermally treated HA-TiN coating at various immersion times. (**a**) Before immersion, (**b**) 1 day, (**c**) 3 days, (**d**) 5 days, (**e**) 7 days, (**f**) 14 days, (**g**) 21 days, (**h**) 28 days, and (**i**) 56 days.

SEM images (Figure 7) of HA-TiN coatings after hydrothermal treatment (Figure 7a) revealed a rough surface with uniform small grains. The cracked line can be observed as a defect on the coating layer. After immersion in the SBF solution, the novel complex is noticeable on the first immersion day (Figure 7b). These complexes increased in number with time. The loosening of the coating, which represented degradation in the coating, can be first noticed on the third day after immersion (Figure 7c). After 14 days of immersion and degradation, more obvious signs correspond with a decrease in the density of the coating (Figure 7f). The coating seemed to loosen when immersion time increased and the precipitation complex increased in number with a smaller diameter. The coating remained, until finished, 56 days after immersion (Figure 7g–i).

#### 3.1.4. Surface Roughness of Coatings

**Figure 8:**
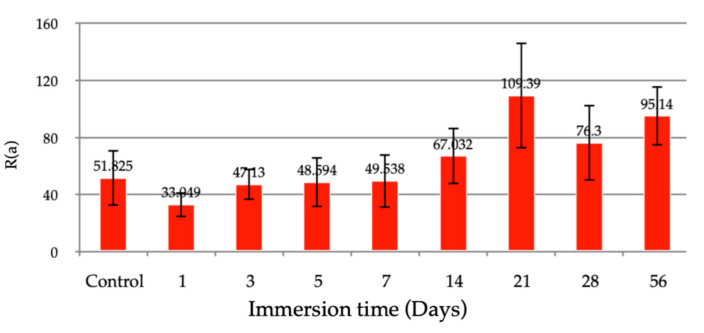
The average surface roughness of hydrothermally treated HA-TiN coating at various immersion times (error bar = 1 SD).

**Figure 9:**
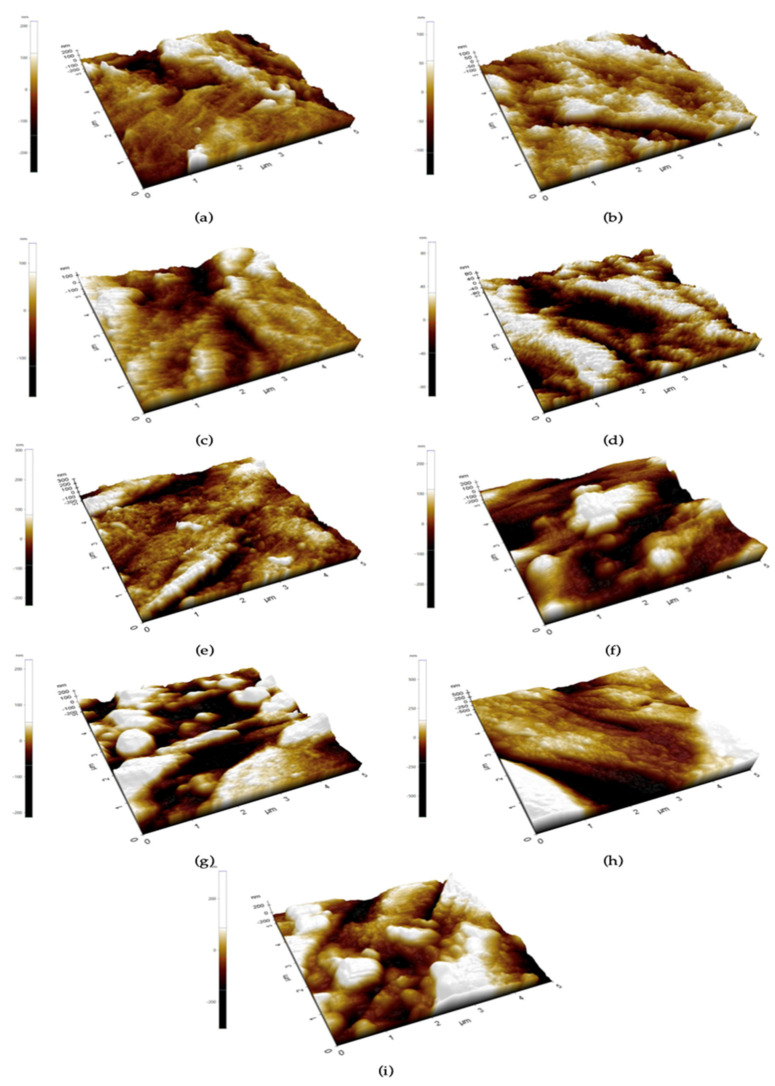
The 3D images of the surface of hydrothermally treated HA-TiN coating at various immersion times. (**a**) Before immersion, (**b**) 1 day, (**c**) 3 days, (**d**) 5 days, (**e**) 7 days, (**f**) 14 days, (**g**) 21 days, (**h**) 28 days, and (**i**) 56 days.

The average surface roughness of the hydrothermally treated coating was 51.78 nm. In the first week of immersion, the surface roughness of the coating was smoother on the first day, then the roughness increased on the third day of immersion and became stable until the end of the first week. Surface roughness increased in the first to the second week and seemed stable after immersion for two weeks, as shown in Figure 8. AFM results confirmed the rough surface of the coating layer. The 3D images (Figure 9) show small grain uniformity on the surface and some of the valleys that might be cracked, as seen in SEM.

### 3.2. Coating Dissolution Behavior

#### Calcium and Phosphorus Concentration

**Figure 10:**
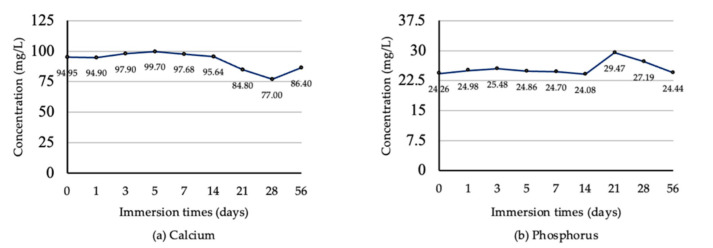
The concentrations of calcium (**a**) and phosphorus (**b**) in SBF solution at various immersion times.

Calcium concentration was stable on the first day of immersion and slightly increased from the third to the fifth day. Calcium concentration started to decrease on the 7th day and continuously decreased until the 28th day of immersion. After day 56 of the immersion test, the concentration was increased (Figure 10a). Phosphate concentration was slightly changed during the first 14 days of immersion. However, the phosphate concentration was distinctly increased on the 21st day of immersion, and then it continuously decreased from the 28th to the 56th day (Figure 10b).

## 4. Discussion

Although PEEK is an alternative material used in orthopedic and dental appliances [1,2,3,4,5,6], the bio-inertness of this material is a significant limitation [6,7]. The present study aimed to investigate the bioactivity of the PEEK surface coated with hydrothermally treated HA -TiN films produced by pulsed DC magnetron sputtering. This was achieved by focusing on their in vitro dissolution, affecting both the physical and chemical properties, especially the Ca/P ratio. The SBF immersion was established to demonstrate when coatings were used in the human body. The limitation of this study is that the static concentration of SBF solution cannot represent the real human body, which can react to events, such as the decrease in local pH at the implantation site before returning to physiologic pH later [14]. These mechanisms may alter the effect of dissolution and mineralization of the coating. Therefore, an in vitro test, simulating the natural environment of the implant site, or an in vivo experiment, is needed to clearly explain the dissolution behavior of the coating.

The Ca/P ratio for the hydrothermally treated coating from the present study (1.28) was lower than our previous study (1.71) [27]. The differences in the power supply system and limited discharge power may be responsible for this. The lower discharge power level caused a lower ionization of argon gas and resulted in a lower deposition rate of the sputtered coating. Additionally, the phase composition of the HA coating was affected by the discharge power [31,32]. Furthermore, the difference in the sputtering parameters, e.g., pulse frequency and argon flow rate, can affect the coating properties [17,33,34]. Although the coating obtained from our studies had lower Ca/P, the coatings proved the toleration to dissolution in SBF with favorable properties for osteointegration throughout 56 days. Moreover, there is evidence from ICP-OES that confirmed the releasing of calcium and phosphate throughout the investigating period.

The XPS results confirmed the formation of HA, while the peaks related to TiN were not shown. The formation of TiO_2_ instead of TiN can be described by the enthalpy of TiO_2_ (−938.72 kj/mol), which is much lower than TiN (−337.65 kj/mol) [35]. The lower enthalpy of compound formation makes that compound more stable than a higher compound that has a higher enthalpy of formation. The coating may not provide a high hardness due to the absence of TiN. However, the presence of TiO_2_ shows benefits in good biocompatibility, bioactivity, and corrosive resistance properties [6,36]. A previous study found TiO_2_ coated on PEEK to promote better osteoblast compatibility. The TiO_2_-coated PEEK showed higher cell adhesion, proliferation, and differentiation than the bare PEEK [37].

The crystallinity in the coatings is a crucial factor in resisting degradation and improving the interfacial bond strength with the substrate [22,23,24,38]. XRD revealed the coatings were in an amorphous phase and only slightly increased HA crystallinity after being hydrothermally treated. Although there was concern about the high solubility of amorphous HA, which can affect the stability of implants, the degradation of the coating was needed to induce the precipitation of bone to promote healing and faster bone fixation [28]. A previous study revealed that a significantly greater bone-to-implant contact was found with the as-sputtered (amorphous) CaP implant in the early period after implantation, corresponding with the pull-out strength of the amorphous CaP coating group, which was higher than others in all investigating times [28]. In addition, a previous study also found that low dissolution of the HA coating had limited bone activity around the implant surface [39]. These studies indicated that even the stability of the coating might affect the implant stability. In contrast, a degradable coating may enhance bone formation and increase the implants’ stability in another way.

The stoichiometric Ca/P of HA is 1.67. However, obtaining an exact stoichiometric ratio is difficult due to methods and conditions after synthesis, which can alter the HA phase [40]. Although the Ca/P was low in some periods of immersion time, the coating remained until the study was completed (56 days), which might diminish the significant limitation of PEEK’s low surface energy. The results of this study revealed the coating method and post-treatment that can obtain suitable Ca/P ratio coatings, which may hasten the bone remodeling when used. A previous study demonstrated that a greater Ca/P ratio of up to 2.5 could increase osteoblast adhesion [41]. Although Lui et al. stated that a Ca/P ratio of 1–2 was an optimum condition for osteoblast viability and could promote osteoblast activities, the osteoblast viability and activity increase was also found in Ca/P at 0.5 and 0.75 [42]. From the literature, the Ca/P we obtained after hydrothermal treatment (1.28) and after being immersed for 56 days (1.16) was in the optimum range in the osteoconductive property.

By combining the atomic percentage from XPS analysis, the Ca and P concentration from ICP-OES analysis, and SEM images, we found that the dissolution and precipitation of the Ca/P complex was a dynamic process, as mentioned before [43]. We found that the trend of dissolution in our study was like the dissolution of plasma-sprayed HA treated with 120 °C vapor for 6 h, which was immersed in SBF, as in the previous study [30]. The results from these dynamic processes made the coating smoother due to the dissolution process in the first week of immersion. This can be observed in the increased Ca and P ions in the SBF solution. In contrast, the atomic percentage of coating components decreased. The coatings became rougher when precipitation dominantly occurred after immersion for two weeks. This corresponded with the drop in both ions’ concentration in SBF after immersion for 14 days. These changes increased the roughness of the coating surface.

The ICP-OES result showed the fluctuance in Ca concentration in SBF in a range of 1.92–2.48 mM, found during the immersion for 56 days. A previous study revealed that Ca concentration in a range of 2–6 mM was suitable for survival and differentiation in mouse primary osteoblast [44]. These indicate that the benefits of the calcium released from these coatings can provide an optimum environment for bone formation. These results from the dynamic process of dissolution and precipitation could be beneficial for cellular and tissue response [45,46]. The effects of these changes in coating topography and Ca concentration may need investigation in future studies.

After being immersed for 56 days, the coating remained with loose density, as seen in SEM. A previous study found that the adhesion strength of the hydrothermally treated HA-TiN coating was stable for 56 days in SBF solution [47], which corresponded with the fact that our coatings remained until the end of immersion at 56 days.

Recent studies showed that PEEK could be a biomaterial for orthopedic and trauma implants, with excellent biocompatibility, and overcomes titanium due to radiolucent properties that facilitate the radiographic follow-up [1,3,4]. The systematic review comparing the titanium and PEEK cages for interfusion showed no difference in postoperative complications. The titanium cage had higher subsidence than the PEEK cage, but the PEEK was lower in fusion rate [48]. Integration between PEEK and bone is essential to these applications’ success. Although unmodified PEEK shows less osteoconduction than titanium [49], surface modification with various bioactive materials can improve the bioactivity of PEEK [50]. Our findings on the chemical properties and integrity of these HA-TiN coatings are a method for enhanced bone remodeling at the PEEK surface, which started on the twenty-first day after implant placement [51]. However, the biocompatibility and bioactive properties of these coatings need to be determined.

## 5. Conclusions

Within the limitations of this in vitro study, SBF immersion immediately affected the physical and chemical properties of the hydrothermally treated HA-TiN coating. The dynamic process of dissolution and precipitation throughout the 56-day immersion period caused physical alteration in the HA-TiN coating and reduced the Ca/P ratio to 1.16. However, the HA-TiN coating remained on the substrate. According to this study, HA-TiN coating via pulsed DC magnetron sputtering, followed by hydrothermal treatment, provided the promising possibility of material bioactivity improvement without disturbing the properties of PEEK. This technique could be beneficial in orthodontics and dental applications.

## Figures and Tables

**Table 1:** Properties of cortical bone, titanium, and orthopedic PEEK [2,16].

Material	Tensile Strength (Mpa)	Elastic Modulus (GPa)
Cortical bone	104–121	14
Titanium PEEK-OPTIMA (LT1) [8]	954–976 100	102–110 4.1

Adapted from Ref. [2], 2019, Carol Davila University Press.

**Table 2:** The ionic concentration (mM) of SBF compared to human body plasma [30].

Solution	Na^+^	K^+^	Ca^2+^	Mg^2+^	HCO^3−^	Cl^−^	HPO_4_^2−^	SO_4_^2−^
Human body plasma	142.0	5.0	2.5	1.5	27.0	103.0	1.0	0.5
SBF	142.0	5.0	2.5	1.5	4.2	147.8	1.0	0.5

Reprinted with permission from [30], 2003, Elsevier.

## Data Availability

The data that support the findings of this study are available on request from the corresponding author.
